# The Allergic Heart That Falls Apart: Type 1 Kounis Syndrome Following Concomitant Diclofenac and Ondansetron Intake

**DOI:** 10.7759/cureus.112179

**Published:** 2026-07-06

**Authors:** Kratika Jain, Gomathi Karmegam, Mithun Manohar, Aarav J Paul, Sahasyaa Adalarasan

**Affiliations:** 1 Emergency Medicine, Madras Medical College, Chennai, IND; 2 Medicine, Madras Medical College, Chennai, IND

**Keywords:** acute coronary syndrome, diclofenac, dyspnea, kounis syndrome, myocardial infarction, ondansetron

## Abstract

Kounis syndrome is an underrecognized cause of acute coronary syndrome characterized by the coexistence of allergic reactions and myocardial ischemia. We report a case of Type 1 Kounis syndrome in a 51-year-old male who presented with acute chest discomfort, diaphoresis, and dyspnea following ingestion of diclofenac and ondansetron. Initial evaluation revealed transient anterior wall ST-segment elevation (V2-V5) with normal cardiac biomarkers and no regional wall motion abnormalities. The patient was initially managed for acute coronary syndrome; however, rapid resolution of symptoms and electrocardiographic changes, along with normal echocardiography and coronary angiography findings, suggested an alternative diagnosis. Clinical features, including GI symptoms without cutaneous manifestations, further supported an atypical allergic presentation. He was treated with intramuscular epinephrine, antihistamines, and corticosteroids, with prompt clinical improvement. A diagnosis of Type 1 Kounis syndrome secondary to drug-induced coronary vasospasm was established. This case underscores the importance of early recognition of Kounis syndrome in acute coronary presentations, particularly in the absence of biomarker elevation and obstructive coronary disease.

## Introduction

Acute coronary syndromes (ACS) comprise a spectrum of conditions precipitated by a sudden reduction or interruption of blood flow in the coronary arteries. The most common cause is rupture or erosion of an atherosclerotic plaque, resulting in coronary artery occlusion. Three forms of ACS are recognized: unstable angina, non-ST-segment elevation myocardial infarction, and ST-segment elevation myocardial infarction (STEMI) [1,2]. Another condition, however, is related to ACS and is similar in clinical presentation but differs in having a multifactorial etiology; this condition is known as Kounis syndrome [2,3].

Kounis syndrome, also termed “allergic acute coronary syndrome” or “allergic myocardial infarction,” is a well recognized but highly underdiagnosed entity [4,5]. First described by Kounis and Zavras in 1991, it represents the coexistence of acute coronary syndrome and an allergic reaction that initiates the episode [1]. Many factors, including drugs and food items, have been associated with triggering allergic reactions [2,4].

Mast cell activation is the key mechanism involved and may be IgE-mediated or IgE-independent [2,4]. This activation triggers the release of multiple inflammatory mediators, such as histamine, leukotrienes, prostaglandins, and platelet-activating factor. The syndrome is classified into three categories: the first is characterized by pure coronary vasospasm in patients with no history of coronary artery disease, the second is associated with preexisting atherosclerotic plaques, and the third involves coronary stent thrombosis [2,4].

The condition is relatively rare, with Type 1 being the most common and Type 3 the rarest form. Patients typically present with a combination of allergic features, such as urticaria, rash, and bronchospasm, as well as cardiac manifestations that mimic myocardial infarction [3,4]. In this report, we describe a middle-aged male who was transferred to our institution from a nearby private hospital as a suspected case of ACS/STEMI precipitated by an atypical allergic reaction.

## Case presentation

A 51-year-old male was referred to the ED of this hospital from a local private hospital as a case of ACS/STEMI. He had been admitted there with complaints of chest discomfort associated with diaphoresis and dyspnea for nearly two hours.

**Figure 1:**
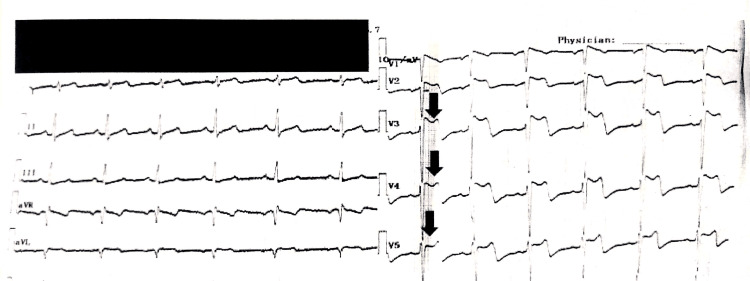
ECG taken before admission Heart rate is within the normal range. The anteroseptal leads show ST-segment elevation mimicking myocardial infarction (marked by the arrows).

He was given a loading dose of 325 mg Ecosprin, 300 mg clopidogrel, and 80 mg atorvastatin for suspected myocardial infarction. An ECG performed at the referring hospital showed ST-segment elevation in leads V2-V5 (anteroseptal leads) with reciprocal changes and a heart rate of 74 beats/min (Figure 1). However, a troponin I test performed at the same time was negative.

On arrival at our hospital, the patient’s airway was patent, and he was able to speak in full sentences. Bilateral air entry was equal, with no added sounds. His oxygen saturation was 98% while receiving 6 L/min of oxygen via an oxygen mask. His respiratory rate was mildly elevated at 22 breaths/min. Circulatory parameters, including blood pressure, pulse rate, and capillary refill time, were normal. His Glasgow Coma Scale score was E4V5M6, and both pupils were equal and reactive to light.

At presentation, there was no history of central left-sided chest pain, compressive pain, or radiating pain of any nature. However, he did complain of giddiness, abdominal pain, and vomiting. There was no history of fever, cold, cough, or diarrhea. Other important differential diagnoses were ruled out by appropriate history and investigations. There was also no known history of comorbidities. He was a social drinker who occasionally consumed moderate quantities of alcohol.

**Figure 2:**
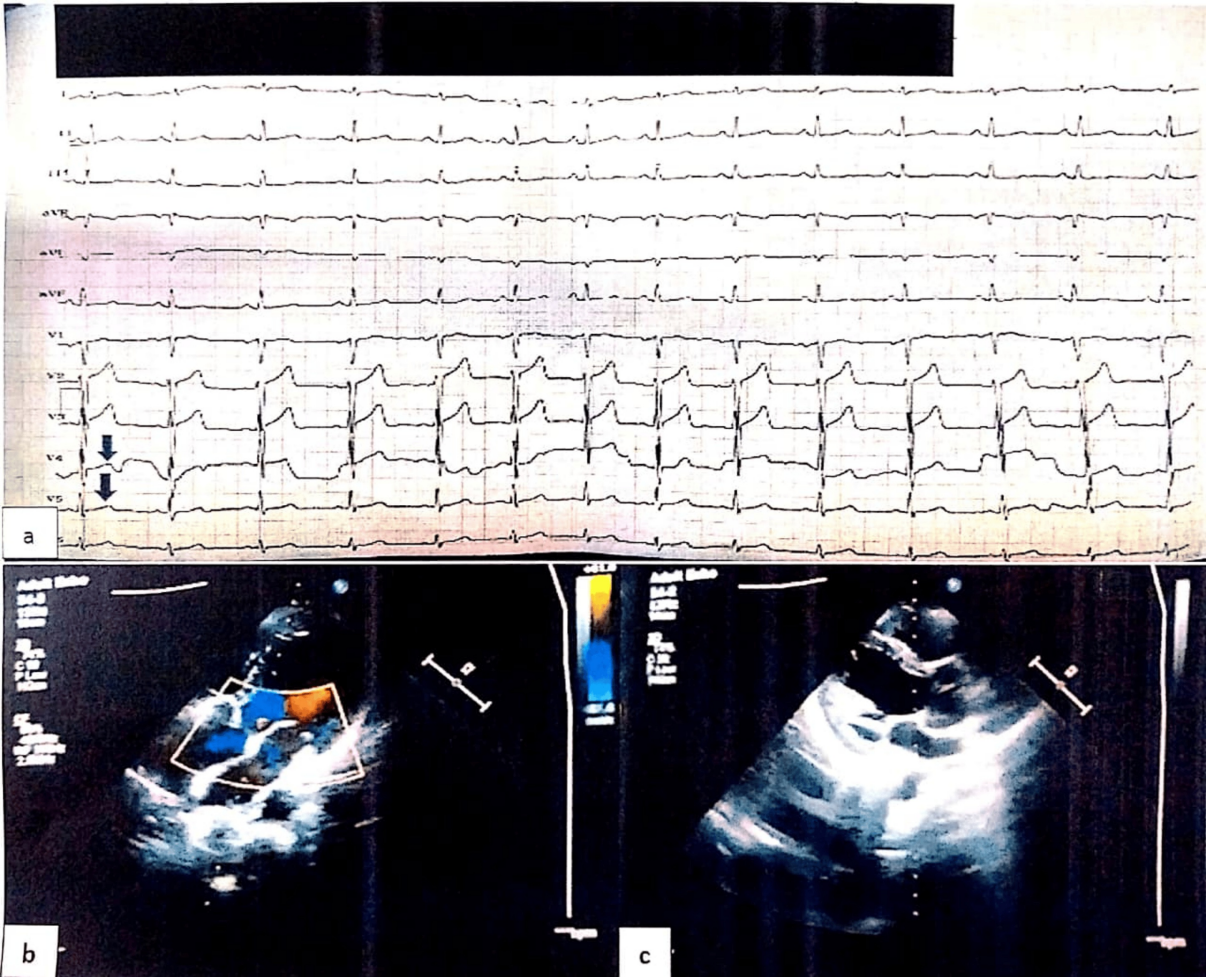
ECG and echocardiogram obtained after admission The figure shows an ECG and echocardiogram obtained four hours after admission. Panel A shows the ECG, with ST-segment changes that had partially returned to normal compared with the initial ECG (marked by the arrows). No other abnormalities were seen. Panels B and C show the echocardiographic findings, which were also normal.

A screening bedside 2D echocardiogram performed in the ED showed no regional wall motion abnormalities, dilated chambers, or valvular abnormalities. The interventricular and interatrial septa showed no thinning, and the ejection fraction was within normal limits. Point-of-care ultrasound showed that the inferior vena cava measured 1.54 cm in diameter and was collapsible, with bilateral lung sliding and A-lines; these findings were not indicative of hemodynamic instability. On further questioning, the patient reported over-the-counter use of diclofenac 50 mg and ondansetron 8 mg for abdominal pain, after which he developed chest discomfort. A repeat ECG and 2D echocardiogram again showed no significant abnormalities, and the ST-segment changes had returned to normal (Figure 2).

Kounis syndrome was suspected, and the patient was treated with epinephrine 0.5 mg IM stat, pheniramine maleate 25 mg IV stat, and dexamethasone 8 mg IV stat, after which a cardiology consultation was obtained. Follow-up coronary angiography was normal. The patient was therefore diagnosed with suspected Type 1 Kounis syndrome, most likely secondary to an allergic drug reaction to diclofenac and/or ondansetron.

## Discussion

Despite growing recognition over the past decade, Kounis syndrome continues to remain a diagnostic challenge in emergency and critical care settings because of its close resemblance to conventional ACS [2,4]. The syndrome should be suspected particularly when ischemic cardiac symptoms occur in temporal association with exposure to a potential allergen, especially in patients lacking traditional cardiovascular risk factors or objective evidence of myocardial injury [3,4].

In the present case, the patient developed acute chest discomfort with transient anterior wall ST-segment elevation shortly after ingestion of diclofenac and ondansetron and ultimately demonstrated normal cardiac biomarkers, normal echocardiography, and angiographically normal coronary arteries. The complete reversibility of electrocardiographic changes following anti-allergic therapy strongly supports the diagnosis of Type 1 Kounis syndrome secondary to transient coronary vasospasm [2,4].

Although myocardial infarction with nonobstructive coronary arteries (MINOCA) should be considered in patients presenting with acute myocardial injury and unobstructed coronary arteries, the diagnosis is one of exclusion and requires evaluation for alternative ischemic and nonischemic etiologies. In the present case, the close temporal relationship between diclofenac and ondansetron administration, the occurrence of hypersensitivity manifestations, and the clinical features consistent with an allergic reaction favored Kounis syndrome over MINOCA. Furthermore, the absence of evidence suggestive of other causes of MINOCA, such as myocarditis, takotsubo cardiomyopathy, coronary embolism, or spontaneous coronary artery dissection, supported this diagnosis.

Among the drugs implicated in Kounis syndrome, nonsteroidal anti-inflammatory drugs (NSAIDs) are frequently reported triggers, with diclofenac representing one of the most commonly associated agents [5]. Wang et al., in a systematic review analyzing 45 published cases of NSAID-induced Kounis syndrome, identified diclofenac as the causative drug in approximately 27% of patients [5]. Chest pain and ST-segment elevation were among the most common presenting features, while a significant proportion of patients demonstrated normal coronary angiograms despite marked electrocardiographic abnormalities. The presentation of our patient closely mirrors these findings [5].

Comparable cases have been documented previously in the literature. Ebrahimi et al. reported a case of acute retrosternal chest pain and dyspnea following intramuscular diclofenac administration. Similar to our patient, transient ischemic ECG changes were noted in the absence of obstructive coronary artery disease, and the patient improved following corticosteroid and antihistamine therapy [6]. A similar scenario was also reported by Rajh et al. [7]. Akboğa et al. likewise described diclofenac-induced anaphylaxis presenting as acute coronary syndrome with normal coronary angiography, further reinforcing the strong association between NSAID hypersensitivity and Type 1 Kounis syndrome [8].

To date, reports of ondansetron-induced Kounis syndrome remain exceedingly rare. One notable case is that of Firew et al., who described a 45-year-old man who developed an anaphylactoid reaction immediately following intravenous ondansetron administration, accompanied by coronary vasospasm and marked troponin elevation [9]. Prior to this, there had been nine documented cases of dysrhythmias and coronary vasospasm associated with ondansetron [9]. To the best of our knowledge, only a small number of cases describing diclofenac-associated Type 1 Kounis syndrome presenting with transient ST-segment elevation and normal coronary angiography have also been reported. After a thorough literature search, we could not identify a reported case implicating both of these drugs simultaneously. Hence, this case is of academic interest with respect to the pathogenetic mechanisms underlying Kounis syndrome and may be the only such reported case to date.

A striking feature in our patient was the transient ST-segment elevation involving leads V2-V5 that subsequently resolved completely on repeat electrocardiography. This dynamic and reversible pattern is characteristic of coronary vasospasm-mediated ischemia and has been consistently observed in Type 1 Kounis syndrome [2]. In contrast to classical STEMI caused by plaque rupture and persistent thrombotic occlusion, the absence of elevated troponin levels and lack of regional wall motion abnormalities in our patient argue against irreversible myocardial injury. Similar findings were observed in the case described by Tang et al., in which flurbiprofen-induced Kounis syndrome produced transient ST-segment elevation despite angiographically normal coronary arteries [10].

The present case also highlights the diagnostic overlap between Kounis syndrome and conventional ACS. The patient was initially treated with dual antiplatelet therapy and statins for suspected STEMI before referral. Such scenarios are common, as emergency management protocols appropriately prioritize rapid treatment of possible myocardial infarction. However, several clues suggested an alternative etiology, including the negative troponin assay, rapid normalization of ECG findings, absence of echocardiographic abnormalities, and the temporal relationship with recent drug exposure [4]. Previous reports have similarly emphasized that delayed recognition often results from failure to elicit a detailed allergy or medication history during the initial assessment, as noted by Ebrahimi et al. [6].

Interestingly, our patient did not exhibit prominent cutaneous manifestations such as urticaria or flushing, which are classically associated with allergic reactions. Instead, GI symptoms, including abdominal pain and vomiting, predominated. This finding is clinically important because atypical allergic presentations may obscure the diagnosis. Wang et al. noted significant variability in allergic manifestations among reported cases, with some patients demonstrating isolated cardiovascular findings [5]. Therefore, the absence of overt dermatological features should not exclude the diagnosis when clinical suspicion remains high.

Management of Kounis syndrome remains challenging because both the allergic and ischemic components require simultaneous consideration. In our patient, administration of intramuscular epinephrine, corticosteroids, and antihistamines resulted in rapid symptomatic and electrocardiographic improvement. Although epinephrine may theoretically aggravate coronary vasospasm through alpha-adrenergic stimulation, its use remains justified in carefully monitored patients with significant allergic manifestations [11]. Similar favorable outcomes with combined anti-allergic therapy have been reported in several previous cases [12].

We were limited by the inability to perform specific investigations such as serum tryptase and serum IgE measurements. We were also unable to conclusively determine whether either or both drugs were responsible for the patient’s symptoms. A more in-depth understanding is needed to clarify the causal role of these drugs in triggering similar allergic episodes with this presentation.

## Conclusions

Kounis syndrome represents an important and often underrecognized differential diagnosis in patients presenting with apparent acute coronary syndrome, particularly when there is a temporal association with potential allergen exposure and rapid reversibility of symptoms or electrocardiographic changes. This case highlights the possibility of the Type 1 variant precipitated by diclofenac and/or ondansetron, manifesting as transient ST-segment elevation with normal cardiac biomarkers and angiographically normal coronary arteries.

The overlap between allergic and ischemic pathways can lead to initial misdiagnosis and conventional ACS management. Early recognition, prompt withdrawal of the offending agent, and combined anti-allergic therapy are crucial for optimal outcomes and prevention of unnecessary invasive interventions. While this condition should be considered, other similarly presenting conditions, such as MINOCA, must also be evaluated. Increased awareness among emergency and cardiology teams is essential to reduce underdiagnosis and improve timely identification of this entity. Reporting such cases further strengthens the existing literature and aids in refining future diagnostic and management strategies.
